# The Effect of Steel and Basalt Fibers on the Shear Behavior of Double-Span Fiber Reinforced Concrete Beams

**DOI:** 10.3390/ma14206090

**Published:** 2021-10-14

**Authors:** Julita Krassowska, Marta Kosior-Kazberuk

**Affiliations:** Department of Building Structures, Bialystok University of Technology, 15-351 Bialystok, Poland; m.kosior@pb.edu.pl

**Keywords:** basalt fibers, steel fibers, shear capacity, cracking, digital image correlation

## Abstract

This study investigates the effects of adding different types of fibers to concrete mixes on the shear behavior of double-span fiber-reinforced concrete beams with or without shear reinforcement. As a part of the experimental study, a total of twenty-seven natural-scale double-span beams were tested. The beams, made of concrete with steel or basalt fiber, with fiber dosages of 78.5 and 5 kg/m^3^, were tested under shear force. The three tested series consisted of three beams with dimensions of 120 × 300 × 4150 mm, with various numbers of stirrups and contents of fiber reinforcement. During the tests, the shear capacity of the elements was determined. The values of support reactions, deflection in the middle of the span of both beam spans, deformations on the surface of the concrete member in the middle of the span in the compressive and tensile zone, and cracking (crack development and crack width) were also measured. The beams were tested using a digital image correlation (DIC) technique. Test results show that shear capacity increases in beams made of concrete with steel (1.87) or basalt fibers (1.23). Moreover, the failure mode changes from shear (brittle) to flexure-shear (less brittle). The experimental shear capacity of beams was compared with the theoretical values predicted by different design codes, i.e., *fib* Model Code 2010 and RILEM TC 162-TDF 2003. The results show that all the design codes underestimate the contribution of fiber-reinforced concrete beams to shear resistance and greatly overestimate the contribution of shear reinforcement.

## 1. Introduction

Double-span reinforced concrete beams are the most frequently used structural members. They are used in elements with large spans or repetitive structures such as slab beams, roof beams, and bridge structures [1]. They are much more economical than single-span beams—their properties are mainly determined by the serviceability limit state, as opposed to the ultimate limit state. Despite the fact that double-span beams are widely used in building engineering, little is known about their behavior under the influence of shear forces. A review of the literature [2,3,4,5] reveals that research works have been mostly devoted to the study of single-span beams under the influence of shear forces, but there is very little reference to double-span beams [6]. Understanding the shear behavior of double-span concrete beams remains a challenge. Furthermore, in the absence of proper shear reinforcement, a sudden shear failure of a double-span concrete beam is likely to occur without any warning, in contrast with the brittle nature of under-reinforced flexural failure. Due to the brittle nature of shear failure, reinforced concrete beams are generally given shear reinforcement to ensure ductile flexural failure. Thus, improving ductility is essential for improved performance and subsequently, fibers can be added to concrete to obtain fiber-reinforced concrete FRC (Fiber-Reinforced Concrete). The addition of steel fibers to enhance the ductility of concrete is not a new idea as it has been practiced over the last few decades [4,7,8]. A completely new solution, however, is the use of basalt fibers. The addition of randomly oriented discrete steel or basalt fibers helps to improve the engineering properties of concrete, i.e., its ultimate strength, post-cracking stiffness, post-cracking tensile strength, and ductility [9,10,11]. Some studies have also shown that conventional (transverse) shear reinforcement can be efficiently replaced with the addition of steel fibers to concrete [5]. This study aims to fill this gap in knowledge by testing the shear behavior of double-span steel or basalt fiber-reinforced concrete beams.

In order to understand the shear behavior of beams, it is first important to understand the transfer mechanism of shear forces. Shear failure of reinforced concrete members results from a complicated mechanism related to the size and type of load, dimensions, the geometric shape of the cross-section, and the material properties of concrete and reinforcing steel. A detailed explanation of the theoretical foundations of the shear phenomenon is given in [12]. The basic mechanisms involved in the transmission of shear force in the oblique section are: aggregate interlock, dowel action, strain softening, axial stress in steel, and the effect of aggregate, always present in ordinary concrete [12].

**Figure 1:**
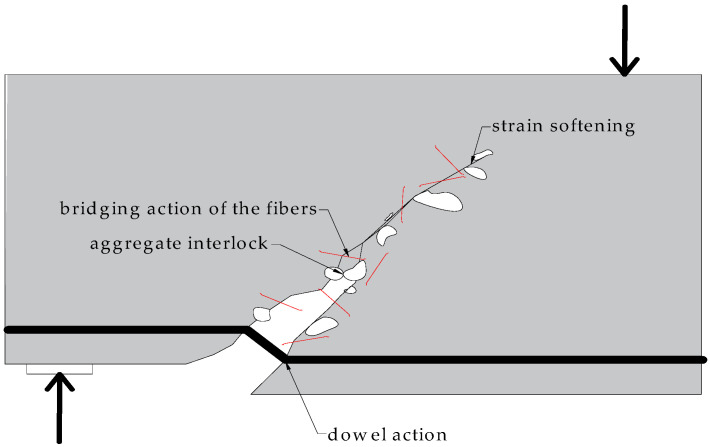
Mechanisms of shear force transmission + fiber influence (own elaboration).

However, these mechanisms only apply to normal concrete and are not relevant to fiber-reinforced concrete (FRC), see Figure 1. This is because in fiber-reinforced concrete, an additional component in the transfer of shear stresses exists, i.e., the bridging action of fibers, which contributes to energy dissipation.

**Figure 2:**
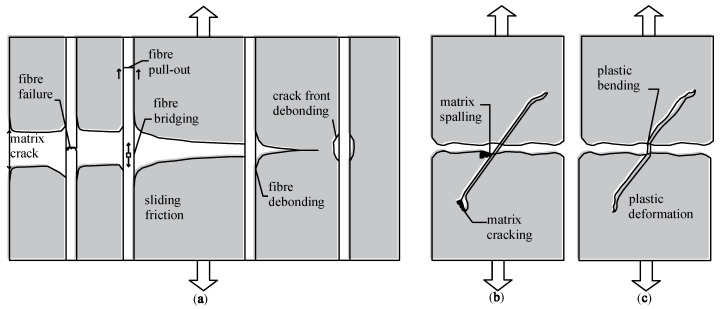
(**a**) A schematic illustration of some of the toughening effects and crack front debonding, the Cook–Gordon effect [14], and debonding and sliding in the crack wake; (**b**) matrix spalling and matrix cracking; (**c**) ductile bending (deformation) of inclined fiber during pull-out—both at the crack and at the end-anchor [13].

This phenomenon was described by Löfgren [13]. The process of fracture of fiber-reinforced composites is a complex phenomenon and depends on a number of parameters. It is generally accepted that when a crack is present in a matrix and it approaches an isolated fiber, the following mechanisms may be expected to take place and contribute to energy dissipation (see Figure 2):

- Matrix fracture and matrix spalling (or fragmentation);
- Fiber-matrix interface debonding;
- Post-debonding friction between fiber and matrix (fibre pull-out);
- Fiber fracture;
- Fiber abrasion and ductile deformation (or yielding) of fibre.

The addition of fibers is one of the methods to alleviate the problem of brittleness in concrete. The principal action responsible for the transfer of shear stresses across a crack in plain concrete is aggregate interlock and friction at the crack faces [15]. For fiber-reinforced concrete, at low and moderate fiber dosages, cracking strength is not affected but, as soon as the matrix cracks, the fibers are activated and starts to be pulled out, resulting in significant toughening behavior [16,17]. For reinforced concrete, the amount of reinforcement crossing the shear plane influences shear friction and shear capacity due to dowel effects; a similar effect is observed for fiber-reinforced concrete beams. The dowel action of fibers was examined by evaluating residual shear stresses at different slip limits and it was found that this increased with the increase in fiber volume fraction. Swamy and Barr reported that shear transfer capacity could be increased significantly, by as much as 60% of the compressive strength [18]. Barragán [4] found that the maximum shear strength increased with fiber volume fraction (for high-strength concrete the increase was significant, close to 100%, with a dosage of steel fibers of 40 kg/m^3^).

The use of steel fibers is a well-known technique [2,7,19]. Furthermore, other types of fibers have appeared, characterized by new properties for which applications are being sought. In the early 1920s, attempts were made to transform basalt rock into basalt fibers. Basalt reinforced polymer bars were then first developed and used as reinforcement in concrete. The addition of chopped basalt fibers to concrete was reported to increase tensile strength and resistance against corrosion; the material is also extremely light and durable [9,10,20,21,22]. The use of corrosion-resistant composites in the production and reinforcement of structural members is expected to increase significantly over the next decade.

Extensive work related to the effectiveness of using fibers as a replacement for stirrups concerns steel fibers [7,23,24]. Nevertheless, very few studies exist that would consider using other types of fibers in this manner [25]. Due to the potential applicability of basalt fibers, further experimental studies should be carried out with a focus on their use in concrete for reinforced structures. The recognition of the failure mode of fiber-reinforced concrete elements is crucial for the development of design methods that would take into account the presence of fiber reinforcement in concrete. On the other hand, the model of behavior of double-span beams is still not sufficiently explained. All tests have been performed on single-span beams. Currently, there is still a lack of study on load-bearing capacity tests of double-span reinforced concrete beams with basalt fibers.

The research presents tests of double-span beams made of innovative concrete with basalt fibers in comparison to beams made of concrete with steel fibers. In this study, the effect of using steel or basalt fibers on the overall shear response and its impact on the ultimate failure mode were examined using the DIC technique. The Digital Image Correlation (DIC) technique is spreading great interest in the research community because of its low costs, effectiveness, and capacity to monitor displacements and strains during experimental tests [26,27]. Hence, the scope of this study is to understand the following:

1. The effect of different fiber materials (steel or basalt fiber) on shear cracking behavior.
2. The contribution of steel or basalt fibers to shear resistance, ductility, and changes in the failure mode of reinforced concrete beams.

## 2. Materials and Methods

### 2.1. Materials

The concrete mixtures were made with CEM I 42,5R Portland cement. The cement content was 300 kg/m^3^ and the *w/c* ratio was 0.5. The aggregate used was a mixture of sand with a grain diameter of up to 2 mm (51% of aggregate) and coarse natural aggregate with a grain diameter of up to 8 mm. Steel and basalt fibers were used as reinforcement in concrete. The steel fibers were the hook-shaped type, 50 mm long with a diameter of 1 mm, tensile strength of 800 MPa, and Young’s modulus of 210 GPa; the diameter of basalt fibers was 20 µm, tensile strength 1680 MPa, and Young’s modulus 90 GPa; these were used to modify the concrete mix. The fiber content in the concrete was 78.5 kg/m^3^ for steel fibers and 5.0 kg/m^3^ for basalt fibers. The fibers were added by volume as a replacement for part of the coarse aggregate. Tests of concrete properties were performed on three series of reference samples and a series of concrete with steel and basalt fibers.

Tests of compressive strength *f_ck_* of concrete were performed pursuant to EN 12390-3:2011 [28] using cubic samples with a side of 100 mm; tests of concrete tensile strength *f_ctm_* on samples with dimensions of 100 × 100 × 400 mm, pursuant to EN 12390-5:2011 [29]; modulus of elasticity *E_cm_* was determined pursuant to EN 12390-13:2014 [30], using cylindrical specimens with a diameter of 150 mm and a height of 300 mm.

The results of tests of the mechanical properties of concrete are presented in Table 1.

The increase in bending tensile strength was 40% for steel fibers introduced into the concrete and 30% for basalt fibers. In the case of concrete specimens with steel fibers, the brittle nature of concrete changed to quasi-ductile. Concrete with basalt fibers exhibited brittle cracking, similarly to plain concrete without the addition of fibers. However, the destructive force was much higher for samples made of concrete with basalt fibers than for plain concrete.

### 2.2. Experimental Program 

**Figure 3:**
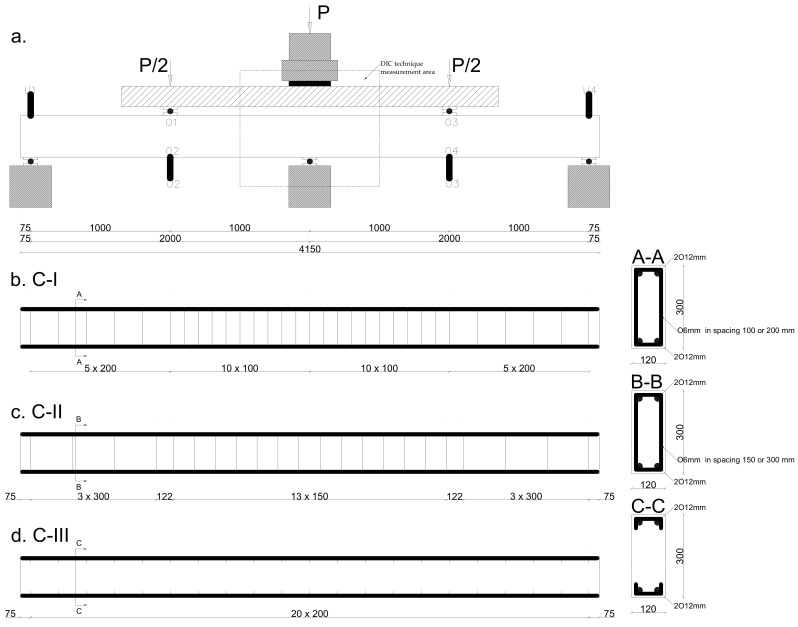
Beam specimen in series (**a**) diagram of measurement points (**b**) C-I, (**c**) C-II, and (**d**) C-III.

Twenty-seven natural-scale double-span beams were tested. Three series of test elements were used, with different types of shear reinforcement. Each series consisted of 3 beams with a dimension of 120 × 300 × 4150 mm each with different numbers of stirrups and three different fiber reinforcement dosages. The series were:

- Series C-I—shear reinforcement with stirrups; maximum spacing determined pursuant to EN 1992-1-1 [31] is 200 or 100 mm, symmetrically with respect to the central support (Figure 3b);
- Series C-II—stirrups with spacing twice as large and the volume is 150 or 300 mm; pursuant to EN 1992-1-1 [31] (Figure 3c);
- Series C-III—without stirrups (Figure 3d).

In each beam series, the proportion of longitudinal reinforcement was $\rho_{e}$ = 0.65%. Additionally, three types of concrete were used in each series: reference W0, WS78.5 with steel fibers in the amount of 78.5 kg/m^3^, and WB5 with basalt fibers in the amount of 5 kg/m^3^.

The beams’ support points were located at a distance of 75 mm from the outer edge of the beams, which resulted in span axes of *l_eff_* = 2000 mm. Shear rate *a_v_/d* of the beams was 3.5.

During the tests, the shear and/or bending capacity of the element was determined. The following values were also measured: support reactions, deflection in the middle of the span of both beam spans, deformations on the surface of the concrete member in the middle of the span in the compressive and tensile zone, and cracking (crack development and crack width).

The beams were preloaded to a force of 30 kN. The time of force buildup was 30 s per 10 kN, and the load was subsequently held constant for 30 s to take the readings of the test values. Then the increment of the force was 10 kN, until failure. Load was applied with a hydraulic cylinder with a capacity of 500 kN, controlled from the control panel of the PZA machine, through a steel traverse and two rollers with a pad directly on the element. The distance between the forces was 2000 mm. The arrangement of the measuring devices on the beam is shown in Figure 3a.

Measurement of the support reactions was performed using Wobit-KMM50 strain gauge force sensors with the following ranges: 0-50 kN and 0-100 kN, with a constant 1.5 ± 2% mV/V. They were placed under the supports in pairs: 2 × 50 kN on the extreme supports and 2 × 100 kN on the central support. The value of force was recorded using the ADAE42U Wobit measuring module connected to force sensors.

The deflection measurements were performed using inductive sensors from the so-called Megatron-Munchen return spring with measuring ranges of 25, 50, and 75 mm, and an accuracy of up to 0.001 mm. The measurements were recorded continuously using the KSR-32 Sensor diagnostic recorder with a sampling frequency of 2 s. The places where the deflections were measured are marked in Figure 3a as U-1/2/3/4. Crack development was also recorded.

**Figure 4:**
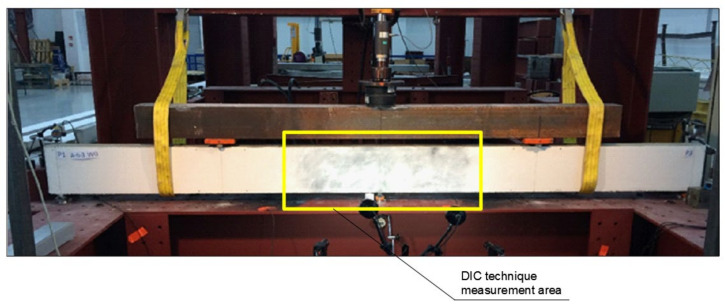
View of the measuring station.

Vision measurement systems used in many industries such as: automotive, automotive design, aviation industry, Bbomechanics/medical, sheet metal forming, casting and foundry and materials testing and simulation. The ARAMIS optical measuring system is designed for non-contact measurements of displacements in planar and spatial elements subjected to loading. The configuration used for the tests, i.e., ARAMIS 2M, is a system consisting of two cameras (Figure 4) with a focal length of 17 mm, a photo speed of 12 Hz, and a resolution of 1600 × 1200 pixels, i.e., 1.92 Mpix. It is a standard set that makes it possible to study the states of displacements and deformations of flat or slightly curved surfaces of spatial elements under a quasi-static load with an accuracy of deformation measurement of up to 0.01% in the image, according to the equipment manufacturer, GOM. In the authors’ opinion, it is more appropriate to provide the accuracy of the reading of position in the image, i.e., 0.01 ÷ 0.04 pixel, depending on the correct calibration of the system.

The accuracy of the reading of position on the real object depends on the measurement range and the ratio of the actual size of the examined area to the size of the analyzed image on the matrix.

The measurement set enabled the recording of up to 5 million readings at a frequency of 15 Hz. The measurement procedure consisted of initial calibration of cameras for a given working area, applying a pattern in the form of black dots with the use of a dedicated preparation, and finally recording the displacement of the measurement points over time, i.e., the so-called facets. The size of the working area was 1000 × 840 mm.

The process of bending of each beam was recorded so that two synchronous photographs of the sample were taken every 1 s, together with the value of the corresponding force. After the completion of the study, the images taken were analyzed using the ARAMIS program, which makes it possible to present the results of the analyses in different manners.

The analyses were performed on the basis of the DIC measurements obtained from one side of the specimen. However, it should be noted that the profile of the crack is not uniform throughout the width of the member.

## 3. Test Results and Discussion

**Figure 5:**
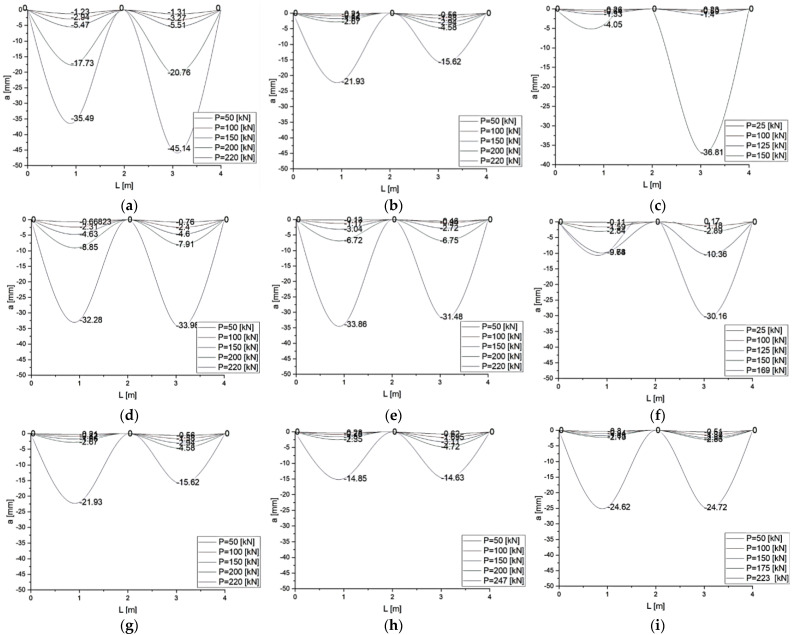
Relationships between force *P* [kN] and mean deflection *a* [mm] in the spans of series: (**a**) C-I-W0; (**b**) C-II-W0; (**c**) C-III-W0; (**d**) C-I- WS78.5; (**e**) C-II- WS78.5; (**f**) C-III- WS78.5; (**g**) C-I-WB5; (**h**) C-II- WB5; (**i**) C-III-WB5.

The maximum measured values of loads, moment capacities, and mid-span deflections; first crack loads, curvature ductility, and failure mode for all the tested beams are compiled in Table 2. The ultimate load is defined as the maximum load achieved by the beam. The load versus mid-span deflection responses of all the twenty-seven beams were plotted as shown in Figure 5. The beams were grouped based on the proposed study parameters. Table 2 lists the number of cracks and the maximum width of perpendicular and diagonal cracks and the inclination angle of the diagonal crack. Crack width was measured just before failure of the beams.

### 3.1. The Effect of Fibers on Capacity 

The effect of fiber reinforcement on shear capacity can be determined by comparing the values of destructive forces in each of the series (Table 2). C-I series beams failed as a result of bending, with the value of the destructive force slightly increased due to the use of steel or basalt fibers in the concrete. The shear failure mode was reached in all C-III series beams. These were not reinforced with stirrups; their role was to be played by fiber reinforcement. When concrete with steel fibers was used, an increase of 87% in the destructive force was obtained, while in the case of concrete with basalt fibers, the increase was 23%. Based on the analyses and subsequent results, it can be concluded that the addition of steel or basalt fibers causes an increase in the destructive force compared to beams without fiber reinforcement.

### 3.2. The Effect on Deflection 

The results of measurements of deflections of reinforced concrete beams are presented in Figure 5. C-I-WS78.5 series beams are characterized by non-symmetrical deflections of spans in relation to the central support. For the C-I-W0 and C-I-WB5 series beams, the values of deflection in both spans were comparable. Both spans of C-II series beams deflected symmetrically, regardless of the added fiber reinforcement. C-III-WS78.5 beams made of concrete with steel fibers deflected symmetrically at both spans, whereas in the C-III-W0 and C-III-WB5 series beams deflection progressed only in the span in which failure occurred.

Adding basalt fibers to concrete caused slight deflections of beams in comparison to C-I series reference elements, whereas adding steel fibers to concrete reduced deflection by as much as a half for the same level of load. Deflections for C-II-WB5 and C-II-WS78.5 series beams were reduced by 30–50% in comparison to the C-II-W0 series. In the case of the C-III series (beam with longitudinal reinforcement without shear reinforcement), the maximum values of deflection of beams made of concrete with steel fibers were 30% lower, while the maximum deflections of beams made of concrete with basalt beams were 20% lower in comparison to deflections of reference beams.

### 3.3. Development of Cracks and Failure Modes of Beams

**Figure 6:**
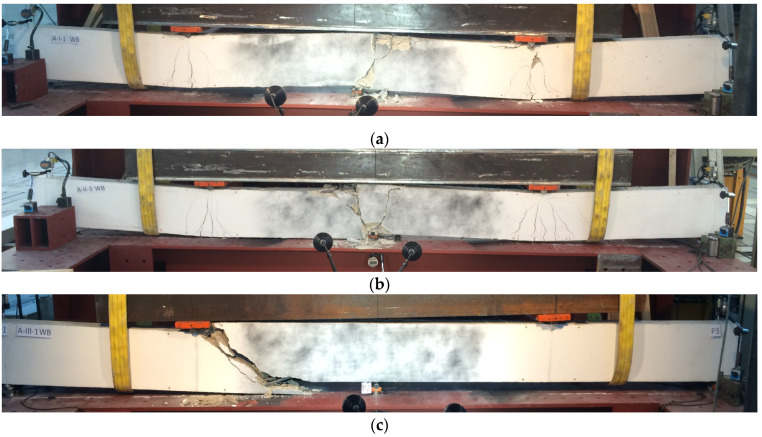
Failure modes for tested beams in series (**a**) C-I-WB5; (**b**) C-II-WB5; (**c**) C-III-WB5.

**Figure 7:**
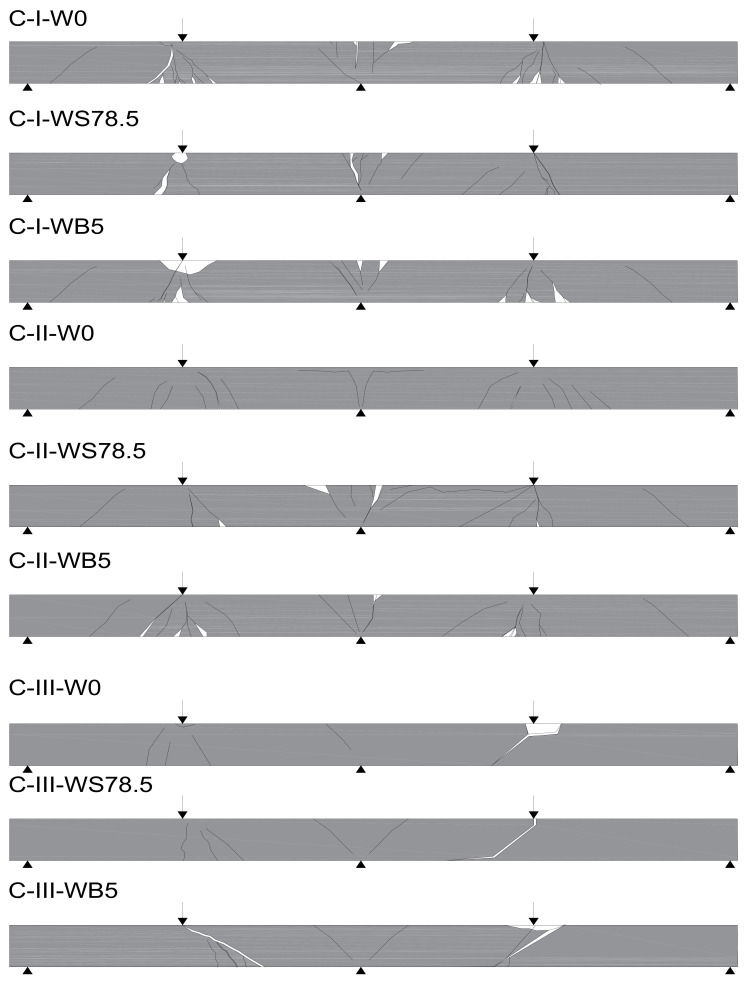
Crack development in tested beams.

During the test, development of cracks in of all the tested beams were monitored as shown in Figure 6 and Figure 7. The main deformations of beams were recorded using the GOM ARAMIS optical data acquisition system—Digital Image Correlation. The characteristic images of deformations at first crack and immediately before failure in the central support area are shown in Figure 9. On the deformation maps, the lighter areas indicate a deeper extent of the tensile zone in the concrete, induced by severe cracks just before failure.

**Figure 8:**
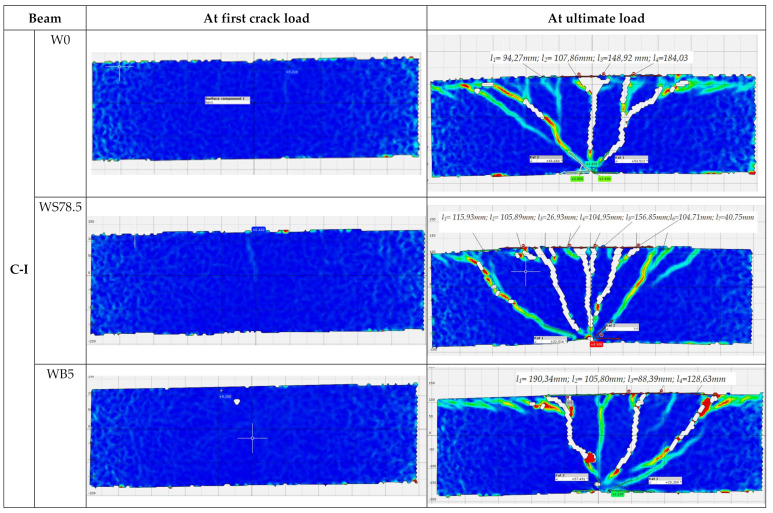
Principal strain contours in the center support in series C-I (*l* is spacing between cracks in mm).

As expected, failure of the C-I series occurred due to bending. In addition, the presence of steel fibers in concrete resulted in the brittle nature of beams changing to quasi-ductile. First cracks in the beams in this series appeared on the central support at a load of 76.7 kN (0.3 *P_ult_*) in the C-I series, at 90 kN (0.4 *P_ult_*) in C-I-WB5 series, and at 86.7 (0.3 *P_ult_*) in C-I-WS78.5 series (Figure 8). As the load increased, new cracks appeared perpendicular to the axis of the element. A redistribution of bending moments could be observed in the beams. Both spans were cracked symmetrically. Failure of reference beams and beams made of concrete with basalt fibers occurred as a result of bending at mid-span and on the central support. In the final stage, failure was quite sudden and its nature was brittle, with the characteristic sound of cracking. C-I-WS78.5 series beams behaved like a ductile object. As the force increased, the existing cracks expanded, but no new ones appeared. At failure stage, the beams were severely cracked at mid-span. The distribution of cracks remained unchanged and its maximum value in the final stage was 184 mm (C-I-W0) in reference beams, and 156 mm (C-I-WS78,5) in beams made of concrete with steel fibers. Beams made of concrete with basalt fibers were characterized by the greatest spacing between cracks on the central support (190 mm). In all the test series, diagonal cracks appeared on the central support; in reference beams and beams made of concrete with basalt fibers, and also at the extreme supports. Diagonal cracks appeared on the central support at the following load levels: C-I-W0–0.7 *P_ult_*; C-I-WB5–0.8 *P_ult_*. In the case of beams made of concrete with steel fibers, diagonal cracks were observed just before failure. The measured angle of diagonal beams on the central support in all C-I beams just before failure was approx. 50° in the direction of the less damaged span (lower stiffness). Later, the beams failed due to clear separation of localized cracks. The number of perpendicular and diagonal cracks as well as the inclination angle of the diagonal crack observed during the test in reference beams were the highest.

**Figure 9:**
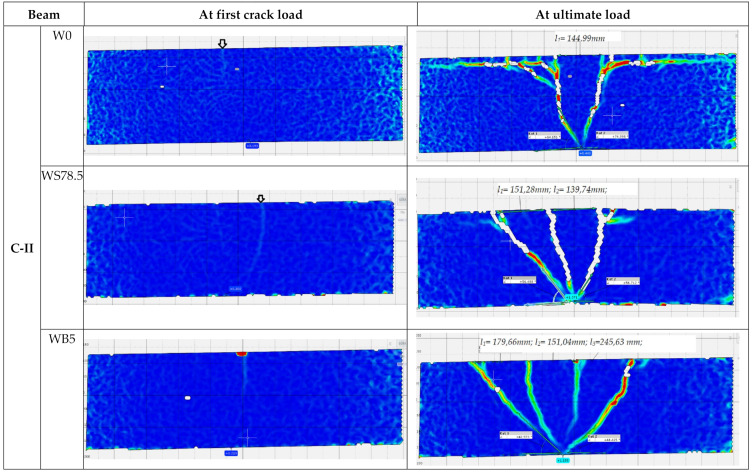
Principal strain contours in the center support in C-II series (*l* is spacing between cracks in mm).

In the C-II series, significant differences in the manner of loss of capacity of the individual elements were observed (Figure 7). Reference beams were cracked first, on the central support at a load level of 0.35 *P_ult_*. These cracks expanded as the load increased and were directed towards the central support (Figure 9). On these beams, the cracking was very symmetrical, both in the spans and on both sides of the central support. At failure stage, cracks were observed along the reinforcement, with the inclination angle for the diagonal cracks of 64° on one side and 74° on the other. Failure occurred as a result of shear on the central support. In C-II-WB5, beams made of concrete with basalt fibers failure also occurred as a result of shear on the central support. The first perpendicular cracks appeared at a force of approx. 0.35 *P_ult_*. In these beams, longitudinal cracks appeared at the upper surface of the beam, stirrup deflections were straightened (at failure stage), in addition to the loosening of the side and upper covers of the reinforcement. Just before failure, diagonal cracks were inclined at an angle of 42° on both sides, while their spacing ranged from 151 to 245 mm. In the C-II-WS78.5 series, failure occurred as a result of bending at the supports. The process of failure was smooth, with significant increase in the deflections in the middle of the spans of the beams at a constant load. During bending, diagonal cracks on the central support reached great widths, with spacings of 151 and 139 mm between them. They did not, however, cause beam failure. In the final stage just before failure, longitudinal cracks appeared along the upper cover. The first crack on the central support was not as destructive in the case of beams made of concrete with basalt or steel fibers in the C-II series.

In the C-I and C-II series, large deformations were observed stretching along the upper cover, connected with the gradual loss of adhesion of the longitudinal reinforcement to concrete. Just prior to failure, the width of cracks was much larger in beams reinforced with steel fibers, while their numbers were smaller than in the case of reference beams, or beams reinforced with basalt fibers. In fiber concrete beams C-II-WB5 and C-II-WS78.5, unlike in the case of C-II-W0 series beams made of reference concrete, local loss of adhesiveness between the cover and the surrounding concrete did not occur. This resulted from the presence of basalt or steel fibers, which transfer tensile stresses.

**Figure 10:**
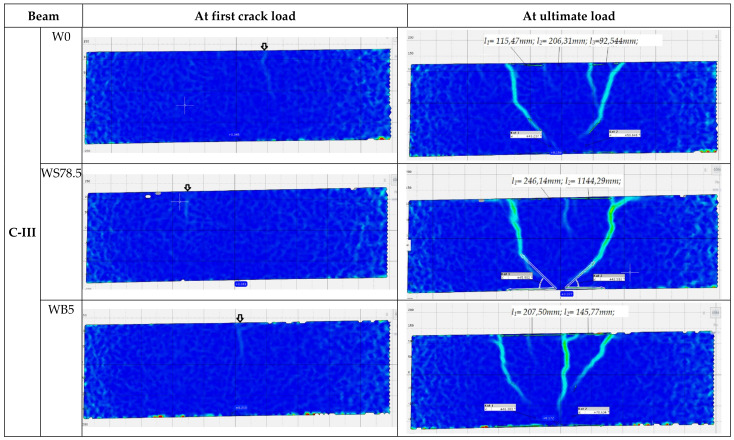
Principal strain contours in the center support in C-III series (*l* is spacing between cracks in mm).

The C-III series without stirrups failed in all cases due to shear. The beams failed at the point where zero momentum and the highest shear force (0.3 *l* from the central support) appear. The first cracks appeared on the central support at the following loads: 0.4 *P_ult_*—C-III-W0, 0.3 *P_ult_*—C-III-W78.5, and 0.4 *P_ult_*—C-III-WB5 (Figure 10). In reference beams and beams made of concrete with basalt fibers, failure occurred suddenly, through shear of the element. In elements in which concrete with steel fibers was used (C-III-WS78.5 series), a stable development of perpendicular cracks up to a height of 0.8 of the height of the beam cross-section was observed. At a load of 0.8 *P_ult_*, a diagonal crack appeared on the central support, which caused failure (Figure 10). One of the beams failed through shear on the central support. In the C-III series, without diagonal reinforcement, deformations in the zone around the supports were small, which is comparable with the pattern of cracking for these beams.

Change in the angle of major shear cracking indicates that the addition of steel or basalt fibers changes the failure mode of reinforced concrete beams from shear (brittle) to less brittle (shear tension failure) with higher displacements at failure.

### 3.4. The Influence of Fibers on Shear Stress

Table 3 presents a comparison of the values of diagonal force causing cracking *V_cr_* and destructive force *V_ult_*. The values shown are for the central support.

The ratio between the diagonal force causing the cracks and the destructive diagonal force turned out to be the highest in the case of beams made referencing concrete C-II-W0, concrete with basalt fibers C-I-WB5, and concrete with steel fibers C-II-WS78.5 at the central support. Diagonal cracks at the central support appeared last in the following series: C-I-WS78.5 (made of concrete with steel fibers), C-II-WB5 (made of concrete with basalt fibers), and C-III-W0 (reference concrete). In the C-II series, with the use of concrete with basalt or steel fibers, diagonal cracks appeared later than in beams made of reference concrete. This points to the effectiveness of using fiber-reinforced concrete to delay the appearance of diagonal cracks. In the C-I and C-III series, at the central support, diagonal cracks appeared faster than in reference beams; this occurred, however, at a higher value of diagonal force. In the C-III series diagonal cracks appeared at the lowest load, at the first stage of beam operation. By using concrete with basalt (C-II-WB5) or steel (C-II-WS78.5) fibers, a *V_ult_/V_cr_* ratio on the central support comparable with C-I-W0 series can be obtained.

The values of shear stresses were determined at three supports for each of the beams. For the C-I series, an increase in shear stresses at the right support was observed. In the case of the left and central support, the stress values were constant. Beams in this series failed as a result of bending in one of the spans, while the other span (less damaged) was still able to transfer loads to the support. In the C-II and C-III series, where beam failure was as a result of shear, the main shear stresses were transferred to the central support. Owing to the use of concrete with basalt fibers, the increase in shear stresses at the central support was 20 and 30% for the C-II and C-III series, respectively.

Regarding shear stresses at the central support, it was found out that in the C-I series, the value of shear stresses increased by 24% owing to the used fiber concrete. Stresses at the extreme supports were considerably reduced, due to the appearance of ductile hinges on the spans, which is related to the fact that the beam started to operate only on the part of the central support with two brackets. In the C-II series, the value of shear stresses increased by 10% due to the redistribution of internal forces and the appearance of a ductile hinge in the C-IIWS78.5 series. In the C-III series, stress values were reduced by 35%. When comparing the values of shear stresses and the course of the support reactions in the C-IIIWS78.5 series, the phenomenon can be explained with redistribution of external forces and the appearance of a ductile hinge in C-IIIWS78.5 support on an external support.

In the C-II series, where failure was due to shear, the main shear stresses were transferred to the central support. The increase in shear stresses on the central support in the C-II series, owing to the use of concrete with steel fibers, was 17%. In the C-III series, a several-fold increase in shear stresses on the external supports was observed. This resulted from transfer (redistribution) of loads to the external supports, thus relieving the central support.

### 3.5. Effect of Fibers on Deformation

**Figure 11:**
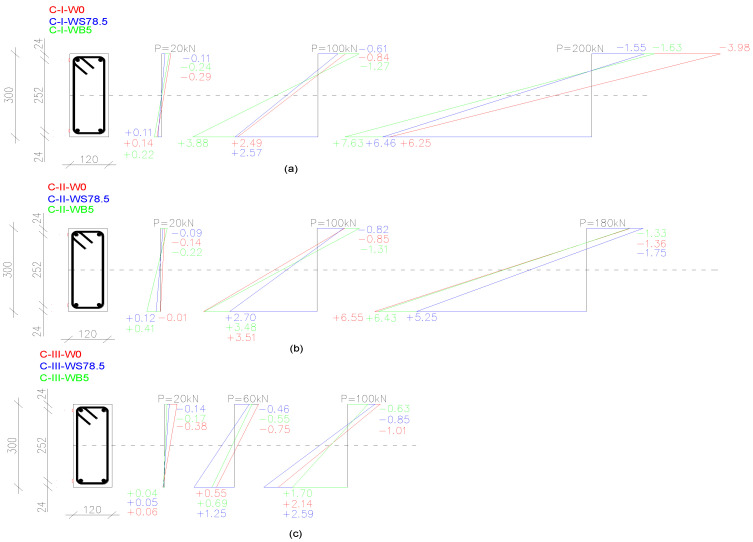
Comparison of average deformations *ε* [‰] at the top and bottom of concrete members at different load levels *P* [kN] in beam series: (**a**) C-I; (**b**) C-II; (**c**) C-III (the lines connecting the points are for reference).

Figure 11 shows the comparative graphs of average values of deformation of concrete on the surface of beams in measurement points at mid-span.

When comparing the values of concrete deformations at the same levels of beam loads, it was concluded that in the C-I series, at the tensile zone, the highest deformations were recorded in the case of beams made of concrete with the addition of basalt fibers during all stages of operation. The difference in deformations reached 56% in comparison to beams from concrete without fibers. The most significant deformations of the compressive zone occurred in the case of reference beams. In the stage before failure, the values were the highest in the compressive and tensile zones in beams made of concrete with basalt fibers.

C-II series reference beams and beams made of basalt fibers were characterized by similar increases in values of deformations both in the compressive and the tensile zone. At a load of 180 kN, beams made of concrete with steel fibers reached the highest values of deformations in the compressive zone (30% and more) and the lowest deformations in the tensile zone (lower by 30%) in comparison to beams made of concrete without fibers.

A similar distribution of deformations can be observed in the C-III series, where reference beams showed the highest values of deformations of concrete in the compressive zone. In the tensile zone, the greatest deformations, at the same level of load, were recorded on the surface of beams made of concrete with steel fibers—approx. 20% higher than for reference beams.

## 4. Capacity Calculations Using RILEM and *Fib*-MC2010 Recommendations 

In order to assess the effect of the presence of fibers on shear capacity, calculations of the load-bearing capacity of beams with longitudinal and fiber reinforcement in the form of basalt or steel fibers were performed, pursuant to *fib* Model Code 2010 [32] and RILEM TC 162-TDF 2003 [33]. The theoretical shear capacity of the beams was calculated based on the above provisions and reported in subsequent sections, with test results reported in [9].

The calculation procedures, taking into account the presence of fibers in concrete, relate to concretes with steel fibers. Due to the lack of guidelines for including basalt fibers in capacity calculations, the calculations employed the same guidelines as that of steel fibers.

The basic problem in the case of concrete with basalt fibers is determining the residual tensile strength at bending. Most methods require a crack mouth opening displacement (CMOD) of 3.5 mm. Concrete with basalt fibers made it possible to obtain deformations corresponding to CMOD values of up to 0.8 mm.

### 4.1. Calculations of Shear Capacity Pursuant to RILEM TC 162—TDF (2003)

The RILEM TC 162–TDF method [33] assumes that the capacity to transfer shear force (*V_Rd_*) is the sum of three components: capacity of concrete member (*V_cd_*), effect of stirrups (*V_wd_*), and effect of fibers (*V_fd_*) [33].

Shear capacity that takes into account the addition of fibers *V_f_* is calculated according to the following Equation:

(1) $$V_{f}=k_{f}k_{l}\tau_{fd}b_{w}d$$

The decisive value is residual strength *f*_*R*,4,_ whose value for CMOD is 3.5 mm, which takes into account residual tensile stresses at bending, determined during testing in conditions of three-point bending of a notched beam.

The shear capacity of beams tested in this study is reported in Section 4.3.

### 4.2. Calculation of Shear Capacity Pursuant to fib Model Code 2010

When tensile fiber reinforcement is planned and members without longitudinal or diagonal reinforcement are considered, main tensile stress *σ*_1_ cannot be lower than the calculated tensile strength of fiber concrete according to:

(2) $$\sigma_{1}\leq \frac{f_{Ftuk}}{\gamma_{f}}.$$

where: *f_Ftu_* is the characteristic value of residual tensile strength of fiber concrete determined for *w_u_* = 1.5 mm.

Equation (3) is applied to steel fiber concrete. The Equation has not been tested for fibers made of other materials and concretes diverging from ordinary ones, such as those made of RPC reactive powders, among others.

(3) $$V_{Rd,F}=\left\{\frac{0.18}{\gamma_{c}}k{\left[100\rho_{1}\left(1+7.5\frac{f_{Ftuk}}{f_{ctk}}\right)f_{ck}\right]}^{1/3}+\sigma_{cp}\right\}b_{w}d$$

Shear capacity *V_Rd,F_* is assumed to be *V_RdFmin_* or higher, determined as:

(4) $$V_{Rd,F\min}=\left(\nu_{\min}+0.15\sigma_{cp}\right)b_{w}d,$$

The model recommends the following method of calculation of shear capacity:

(5) $$V_{Rd,F}=\frac{1}{\gamma_{F}}\left(k_{v}\sqrt{f_{ck}}+k_{f}f_{Ftuk}cot\theta \right)b_{w}z,$$

(6) $$k_{v}=\frac{0.4}{1+1500\varepsilon_{x}}\cdot \frac{1300}{1000+k_{dg}z}\;for\;\rho_{w}<0.08\sqrt{\frac{f_{ck}}{f_{yk}}},$$

(7) $$k_{v}=\frac{0.4}{1+1500\varepsilon_{x}}\;for\;\rho_{w}\geq 0.08\sqrt{f_{ck}/f_{yk}}.$$

Shear capacity of beams with longitudinal or diagonal reinforcement should be calculated according to the following Equation:

*V_Rd_* = *V_Rd,F_* + *V_Rd,s_* (8)

The shear capacity of the beams tested in this study is reported in Section 4.3.

### 4.3. Comparison of Results 

**Figure 12:**
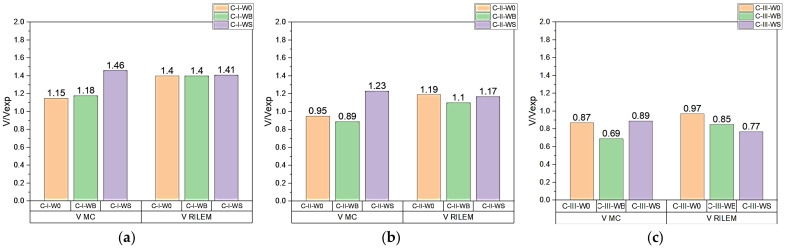
A comparison between experimental force *V_Exp_* [kN] and theoretical calculations performed pursuant to *fib* Model Code 2010 (*V_MC_*) and RILEM TC 162-TDF 2003 (*V_RILEM_*) for series: (**a**) C-I; (**b**) C-II; (**c**) C-III.

Table 4 and Figure 12 show the ratio between shear capacity calculated pursuant to *fib* Model Code 2010 (*V_MC_*) and RILEM TC 162-TDF 2003 (*V_RILEM_*) in relation to the value of diagonal force determined experimentally *V_Exp_*.

C-I series beams failed due to bending, which is why the calculated capacity values were higher than the diagonal force determined experimentally.

C-II series beams failed mainly as a result of bending, then due to shear at the central support. The radio between the calculated and the experimentally determined shear capacity at the central support should thus be considered. All calculation procedures correctly forecast the values of diagonal force. In the case of beams with longitudinal or diagonal reinforcement, and/or fibers, shear capacity mainly depends on the shear capacity component as a result of the diagonal reinforcement. The addition of steel fibers increases the value of shear capacity by 18%.

In series C-III, failure occurred at the central support. The calculated values of capacity, regardless of the estimation method, were lower than those obtained experimentally, despite the fact that the safety coefficient was not included in the performed calculations. This means that each of the methods assume a reserve of capacity. The values of forces determined experimentally were higher, depending on the calculation method, by 11–43% for *fib* Model Code 2010 and 3–23% for RILEM TC 162-TDF 2003. In the case of the C-III-WB series, regardless of the calculation method, a reserve of capacity of up to 20% was obtained. In the case of the C-III-WS series, the method that reflected the experimental value of the destructive force was the *fib* Model Code 2010 procedure.

**Figure 13:**
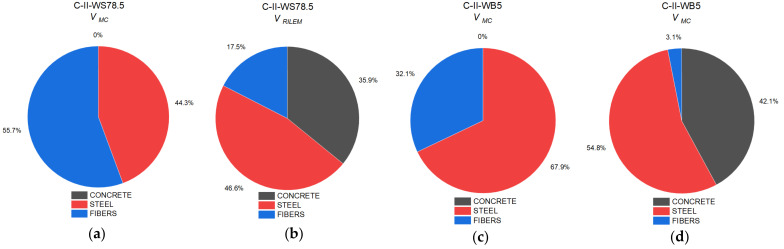
Proportion of the individual components of shear capacity of beams in the following series: (**a**) C-II-WS78.5 pursuant to *fib* Model Code 2010; (**b**) C-II-WS78.5 pursuant to RILEM TC 162-TDF 2003; (**c**) C-II-WB5 pursuant to *fib* Model Code 2010; (**d**) C-II-WB5 pursuant to RILEM TC 162-TDF 2003.

Figure 13 shows a comparison of the proportional role of capacity as assigned to concrete cross-section, longitudinal reinforcement, and the presence of fibers in the total shear capacity of series with basalt or steel fibers. Beams made of concrete with steel fibers were characterized by a higher value of shear capacity than beams made of concrete with basalt fibers. The basic parameters in Equations for the calculation of shear capacity of fiber concrete is residual tensile strength at bending, which in the case of concrete with steel fibers is much higher than that for concrete with basalt fibers.

**Figure 14:**
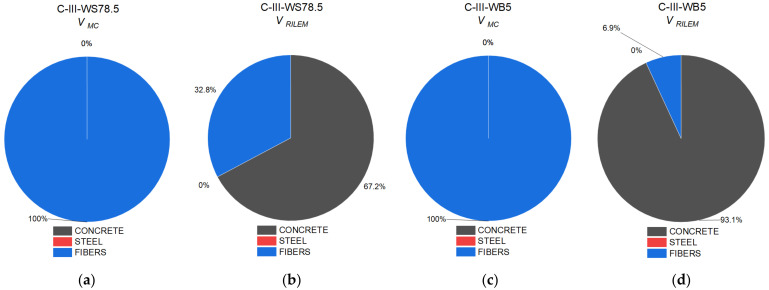
Proportion of the individual components of shear capacity of beams in the following series: (**a**) C-III-WS78.5 pursuant to *fib* Model Code 2010; (**b**) C-III-WS78.5 pursuant to RILEM TC 162-TDF 2003; (**c**) C-III-WB5 pursuant to *fib* Model Code 2010; (**d**) C-III-WB5 pursuant to RILEM TC 162-TDF 2003.

Figure 14 shows the proportion of components in shear capacity depending on the force transferred by the concrete cross-section and the proportion of fiber reinforcement. The *fib Model Code 2010* procedure [32] contains one Equation that takes into account the impact of fiber reinforcement by including the residual strength of fiber concrete. The increase in shear capacity was 77%, in comparison to beams made of concrete without fibers. In the RILEM TC 162-TDF 2003 procedure [33], the proportion of fibers in shear capacity was included as an additional component. The presence of fibers contributes to an increase in shear capacity in beams made of concrete with steel fibers by 36% in comparison to reference beams. In the C-III-WB5 series, a comparison shows that the inclusion of the impact of basalt fibers in the calculation of shear capacity better reflects the actual load-bearing capacity, yet it still retains a reserve of capacity at a level of approx. 20%.

Owing to the presence of steel fibers, regardless of the calculation method, the component of shear capacity was higher in comparison to concrete with basalt fibers.

## 5. Conclusions

The study aimed to demonstrate the effect of steel fibers in the amount of 78.5 kg/m^3^ and basalt fibers in the amount of 5 kg/m^3^ on the improvement of the load-bearing capacity of double-span beams with a length of *L* = 4.15 m. The effect of the fibers was assessed in comparison to test results for concrete members without fiber reinforcement. Based on the experimental study, the following major conclusions can be drawn:

- The shear capacity of double-span beams *L* = 4.15 m increased in beams made of concrete with steel or basalt fibers.
- The shear stresses at the center support were greater compared to the volume of stresses at the extreme supports. The volume of shear stress depends on the degree of shear reinforcement (the greater the degree of shear reinforcement, the greater the value of shear stresses). The presence of fiber reinforcement in the concrete also increased shear stresses.
- Beams in all series made of concrete with steel or basalt fibers were characterized by lower deflections compared to reference beams at the same load level. Due to the use of steel fibers in concrete, the values of deflection in all series of beams were three times smaller, while in the case of concrete with basalt fibers in C-III-WB5 beams, they were twice as small compared to beams made of concrete without fibers.
- Change in the angle of diagonal cracking indicates that the addition of steel or basalt fibers changes the failure mode of reinforced concrete beams from shear (brittle) to less brittle (shear tension failure) with higher displacements at failure.
- Digital Image Correlation (DIC) analysis of the crack pattern around the central support confirmed the influence of both types of fibers on the crack pattern and shear behavior of beams with reduced reinforcement for shear.
- The shear capacity of SFRC (steel fiber-reinforced concrete) beams tested in this study, consistent with literature data, were estimated using *fib*-MC2010 and RILEM guidelines. RILEM guidelines resulted in closer predictions of test results. The *fib*-MC2010 recommendations gave a more conservative estimate of the capacities than RILEM provisions.
- In times of rising steel prices they are sought to replace it. The test results confirm the possibility of complete or partial replacement of steel stirrups by concrete with basalt fibers.

## Figures and Tables

**Table 1:** Measured Average Values for Concrete with Various Contents of Fiber.

Fiber Content		*f_ck_*	*f_ctm_*	*E_cm_*
	[kg/m^3^]	[MPa]	[MPa]	[GPa]
W0	0	35.19 (±0.86)	5.14 (±0.33)	33.29 (±1.28)
WS78.5	78.5	39.12 (±4.11)	7.21 (±1.06)	33.71 (±0.34)
WB5	5	29.08 (±2.94)	6.72 (±1.22)	32.86 (±2.33)

**Table 2:** Summary of Test Results for all Beams.

Beam		Max. Load (kN)	Mid-Span Deflection (mm)	1st Crack Load (kN)	1st Diagonal Crack Load (kN)	Number of Diagonal Cracks (-)	Width of Diagonal Crack (mm)	Angel of Diagonal Crack (^°^)	Failure Mode (-)
	W0	222	8.09	76.7	118.3	4	1.6	43.0	Flexural
**C-I**	WS78.5	258	3.61	86.7	220.0	1	1.1	44.7	Flexural
	WB5	229	8.44	90.0	160.0	3	1.9	40.3	Flexural
	W0	193	8.68	66.7	96.7	1	0.7	43.0	Shear
**C-II**	WS78.5	252	3.04	80.0	146.7	3	1.4	40.7	Flexural
	WB5	228	4.54	83.3	136.3	2	1.4	44.7	Shear
	W0	109	5.18	73.3	88.3	2	30.2	42.0	Shear
**C-III**	WS78.5	204	3.82	83.3	123.3	2	23.7	33.3	Shear
	WB5	134	3.13	86.7	91.7	2	28.5	29.0	Shear

**Table 3:** Summary of Average Values of Shear Forces Causing Diagonal Cracking *V_cr_* and Shear Forces *V_ult_* at Maximum Load (at the central support).

Beam		*V_cr_*	*V_ult_*	*V_ult_/V_cr_*
**C-I**	W0	42.4	78.5	1.9
	WS78.5	69.45	97.55	1.4
	WB5	40.95	80.45	2.0
**C-II**	W0	21	47.95	2.3
	WS78.5	32.15	56.05	1.7
	WB5	39.25	57.95	1.5
**C-III**	W0	30.75	36.85	1.2
	WS78.5	27.25	41.85	1.5
	WB5	30.95	48.7	1.6

**Table 4:** Comparison of the Values of Shear Capacity Obtained from Experimental Result (*V_Exp_*) and from RILEM (*V_RILEM_*) and *fib* Model Code (*V_MC_*) Calculations of the Tested Beams.

Beam		*V_exp_*	*V_MC_*				*V_RILEM_*			
			*V_Rd,c_*	*V_Rd,F_*	*V_Rd,s_*	*V_Rd_*	$V_{c}$	$V_{wd}$	$V_{f}$	*V_Rd_*
**C-I**	W0	76.3	17.27	0	70.8	88.1	36.3	70.8	0	107.1
	WS78.5	88.6	0	76.88	70.8	129.72	36.3	70.8	17.68	124.78
	WB5	78.7	0	22.3	70.8	93.1	36.3	70.8	2.7	109.8
**C-II**	W0	70	19.55	0	47.2	66.75	36.3	47.2	0	83.5
	WS78.5	86.6	59.23	47.2	106.43	106.43	36.3	47.2	17.68	101.18
	WB5	78.3	0	22.3	47.2	69.5	36.3	47.2	2.7	86.2
**C-III**	W0	37.4	30.38	0	0	30.38	36.3	0	0	36.3
	WS78.5	70.1	0	0	0	62.53	36.3	0	17.68	53.98
	WB5	46	0	0	0	3.7	36.3	0	2.7	39.0

## Data Availability

Data sharing is not applicable to this article.
